# *Panax notoginseng* Saponins Alleviate Coronary Artery Disease Through Hypermethylation of the miR-194-MAPK Pathway

**DOI:** 10.3389/fphar.2022.829416

**Published:** 2022-06-16

**Authors:** Lian Duan, Yongmei Liu, Jun Li, Yun Zhang, Yan Dong, Chao Liu, Jie Wang

**Affiliations:** Department of Cardiology, Guang Anmen Hospital, Beijing, China

**Keywords:** *Panax notoginseng* saponins, coronary artery disease, DNA methylation, miRNA, MAPK

## Abstract

**Background:** *Panax notoginseng* saponins (PNS) may have an inhibitory effect against coronary artery disease (CAD); however, the mechanism is unclear. Recent research has begun to evaluate the role of epigenetics in CAD. Our team found that hypomethylation of miR-194 could be an important mechanism of CAD.

**Purpose:** The aim of this study was to investigate the effect of PNS against CAD and evaluate whether the mechanism is related to methylation of mi-R194.

**Methods:** We conducted a randomized controlled trial with a double-blind placebo design on 84 patients with CAD. Treatment was continued for 4 weeks, and the clinical effect of PNS on CAD was observed. Methylation of miR-194, its promoter, and the key nodes of the MAPK pathway were measured by pyrosequencing and qRT-PCR. We then conducted a pharmacological analysis of the active components of PNS. The effects of PNS on oxidized human umbilical vein endothelial cells and the methylation of miR-194, its promoter, and the key nodes of the MAPK pathway were measured *in vitro* through methylation-specific PCR (MSPCR), qRT-PCR, Western blot analysis, and annexin V/propidium iodide apoptosis assay.

**Results:** PNS improved symptoms of CAD. High-density lipoprotein and white blood cell count demonstrated significant changes after treatment in the PNS group. No significant difference was observed between miR-194 and mRNA MAPK, FAS, RAS, and FOS in the PNS group after treatment. However, some notable trends were observed in these genes. The targets of PNS were predicted by the pharmacological components. Some targets were found to be differentially expressed genes in CAD sequencing. Six genes, including MAPK1, RAS, and FASL, were common targets of PNS in CAD sequencing. Correlations were observed between genes in the interaction network and clinical parameters. *In vitro* experiments confirmed that PNS could change the methylation of miR-194, its promoter, and MAPK, FAS, RAS, and FOS. Intervention with PNS is likely to improve apoptosis.

**Conclusion:** We reported the regulation of miR-194 promoter, miR-194, and MAPK methylation by PNS through cell experiments and a randomized controlled trial. PNS can be used for intervention in CAD by targeting the miR-194 promoter-miR-194-MAPK signaling pathway.

**Clinical Trial Registration**: https://www.clinicaltrials.gov/, NCT03083119.

## Introduction

Despite massive efforts to develop cardiovascular treatments, coronary artery disease (CAD) remains the leading cause of mortality and morbidity in the world (Douglas et al., 2016). In recent years, traditional Chinese medicine (TCM) has received widespread attention. *Panax notoginseng* (Burkill) F.H.Chen [Araliaceae] is particularly popular among patients with CAD because many studies have demonstrated the inhibitory effects of this plant against CAD. *Panax notoginseng* saponins (PNS) are the main active ingredient in this plant (Duan et al., 2017). PNS has multiple effects on CAD, including anti-inflammation, regulation of lipid metabolism and the coagulation system, anti-apoptosis, pro-angiogenesis, anti-atherosclerosis, and anti-myocardial ischemia (Zhang et al., 2016; Shen et al., 2017; Zhou et al., 2018; Xiong et al., 2020; Wang et al., 2021). Long-term use of PNS can effectively reduce the end point of CAD and improve angina pectoris, electrocardiogram (ECG), and lipid metabolism, illustrating that PNS is a potential agent against CAD (Duan et al., 2018).

Recent research has begun to discover the role of epigenetics in cardiovascular disease development. According to our previous research (Rupérez et al., 2021), miR-194 promoter-methylation-miR-194-mitogen-activated protein kinase (MAPK) could be biomarkers of CAD.

## Materials and Methods

### Participant Recruitment and Sample Collection

The study population consisted of 84 patients presenting within 15 days of an unstable angina (UA) event at Guang Anmen Hospital. Patients were included if they had a coronary angiography to estimate the extent of CAD for all subjects according to the criteria defined by the American Heart Association. Those with at least one major epicardial vessel with >50% stenosis were defined as CAD subjects, while those with <50% stenosis were defined as control subjects (Austen et al., 1975; Zhao et al., 2019). Finally, those aged 45–75 years were included. The exclusion criteria consisted of an index event due to uncontrolled hypertension and/or blood pressure remaining ≥180/110 mmHg despite treatment, New York Heart Association class III or IV congestive heart failure irrespective of ejection fraction, or class II heart failure with left ventricular ejection fraction ≤40% persisting at the end of the run-in period despite treatment, severe valvular heart disease, arrhythmia, cardiomyopathy, stable angina pectoris, clinically apparent liver; kidney; hematological system; nervous system; or mental disease, malignancy (except for nonmelanoma skin cancer) within the preceding 3 years, and inability to provide informed consent or comply with study requirements.

The study protocol was approved by the institutional ethics committee of Guang Anmen Hospital, Beijing, and is registered at *Clinical Trials* (identifier: NCT03083119) (https://www.clinicaltrials.gov/). All patients provided written informed consent.

A standardized questionnaire was applied to assess smoking history, hypertension, and diabetes among study subjects. Smoking history was classified as either “no smoking” or “smoking” (including both former and current smokers). Hypertension status was classified as either “non-hypertension” or “hypertension” (including the previous diagnosis of hypertension and systolic blood pressure (SBP) ≥ 140 mmHg and/or diastolic blood pressure (DBP) ≥ 90 mmHg currently). Diabetes status was classified as either “non-diabetes” or “diabetes” (including previous diagnosis of diabetes and fasting blood glucose ≥7.0 mmol/L or postprandial blood glucose ≥11.1 mmol/L) (Ge et al., 2016).

Blood samples of patients on an empty stomach for 12 h were drawn in the morning within 24 h after admission. Blood of the outpatients and controls was drawn in the morning of the second day after admission. Specifically, 4 ml of venous blood was collected and placed in an EDTA tube. Peripheral blood mononuclear cells (PBMCs) were centrifuged within 6 h.

### Randomization and Blinding

SPSS 19.0 software was used to randomly generate the 1:1 allocation scheme of the subjects. Drugs with corresponding numbers were given, according to the order of treatment. A random card was made to record the distribution plan and then sealed in an opaque envelope. A double-blind design was used in this study, and PNS and PNS placebo were numbered as group 1 or group 2. According to the drug distribution method of double-blind experiments, the drugs were packed in the same box with different drug numbers. The blind bottom was made in duplicate and managed by a specially assigned person. After completion of the trial, the data from group 1 and group 2 were statistically analyzed.

### Study Treatment

**FIGURE 1:**
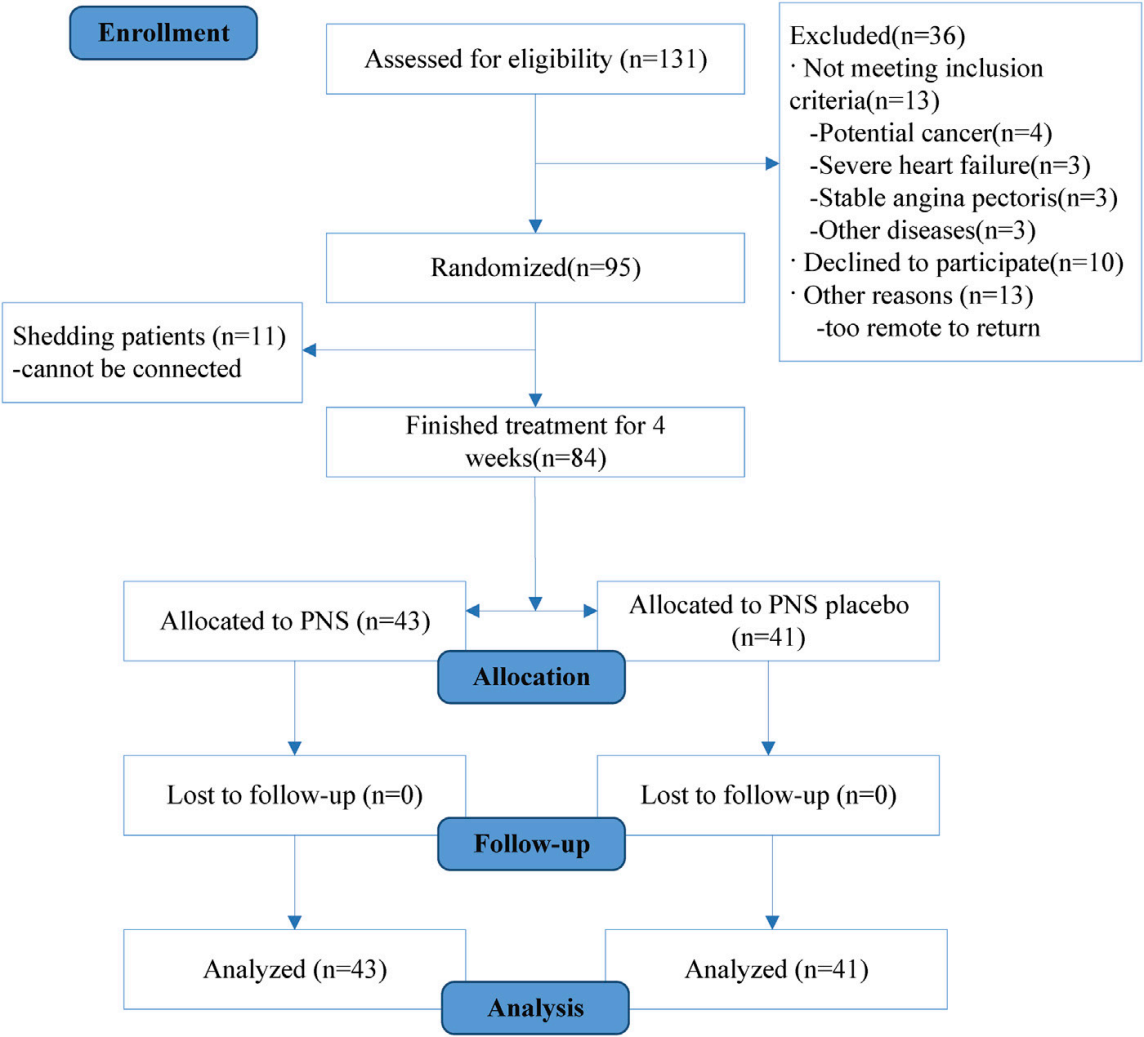
Trial protocol timeline as described in detail in the Materials and Methods section.

This study used a double-blind, placebo design (Xuesaitong soft capsule and Xuesaitong soft capsule placebo). The main ingredient of the Xuesaitong capsule is PNS. The experimental group was treated with Xuesaitong capsules based on conventional drugs, and two capsules (0.66 g) were given twice a day. The placebo group was treated with Xuesaitong placebo capsules (specifications: 0.33 g × 12 s × 2 board, Kunming Torch Pharmaceutical Group Co., Ltd.). Xuesaitong soft capsule or Xuesaitong soft capsule placebo was administered 0.5 h after conventional drugs. Treatment continued for 4 weeks. Treatment was not combined with other Chinese decoctions or proprietary Chinese medicines during the trial. Furthermore, all subjects were strongly recommended to receive other concomitant secondary prevention medications, according to practice guidelines (Roffi et al., 2016). All participants underwent follow-up examinations 28 and 60 days after treatment (Figure 1).

The Xuesaitong capsule (State medical permit Z19990022, specifications: 0.33 g × 12 s × 2 board, Kunming Torch Pharmaceutical Group Co., Ltd.) batch number was 20150417, and each capsule contained 60 mg of medicine. The composition was 5.4 mg R1, 31.8 mg Rg1, and 17.2 mg Rb1. The placeboes were glue pills filled with starch and caramel pigment and had an identical look, color, and weight to XSTSC.

Study treatment was administered on a background of conventional drugs, including antiplatelet, anticoagulant, statin, vasodilator, diuretic, hypotension, and hypoglycemic drugs, and the combination drugs were allowed before and during clinical trials (including emergency treatment drugs) in accordance with the diagnosis and treatment procedures. Drugs prohibited before and during the clinical trial included other Chinese patent medicines and traditional Chinese medicine decoctions not provided in this study.

### Verification of the Methylation Level of Related Sites by Pyrosequencing

The reaction system was set up for bisulfite transformation, which was carried out on a 9700 PCR instrument with a hot cover, and the DNA was frozen and preserved after treatment. For PCR (PyroMark PCR kit) amplification, the single-chain PCR products were purified, and the primers were annealed. The PCR products were retained for pyrosequencing on the PyroMark ID instrument, and the assay was established by PyroMark cytidine-phosphate-guanosine software. The related items were prepared and sequenced, and the results were analyzed.

### Detection of the miR-194 Promoter Methylation Level by Methylation-Specific PCR (MSPCR)

The genomic DNA of tissue or peripheral blood was extracted using the phenol chloroform method. DNA concentration, OD260, OD280, and OD260/OD280 were measured using a nanodrop 1000 spectrophotometer. The DNA sample OD260/OD280 used in this experiment was 1.8–1.9, which was regarded as qualified DNA. The DNA samples were treated with bisulfite, amplified by PCR, and reacted with a self-analysis platform. The 384-pore plate was put into the sample adding instrument. After the parameter setting was complete, the sample could be spotted on the chip. After sampling, the chip was placed in the mass spectrometer, and methylation analysis was performed according to the molecular weight difference of C and T bases in the fragment. The methylation ratio of mass spectrometry was obtained by EpiTYPER software version 1.0 (Sequenom, San Diego, CA, United States).

### Real-Time Fluorescence Quantitative PCR

Cells were collected and treated 72 h later. The total RNA was extracted using the TRIzol method and then reverse transcribed into cDNA for PCR. For PCR, cDNA was diluted 10 times with 4 μL SYBR Green, 5 μL primer, 0.2 μL + primer (10 μmol/L), 0.2 μL each, and 0.6 μL water for a total of 10 μL. PCR was performed at 95°C for 5 min, 95°C for 10 s, and 60°C for 20 s for a total of 50 cycles and then at 5°C for 10 s, 60°C for 10 s, and 40°C for 30 s. The CT values of the target and control genes were automatically collected by using the fluorescence quantitative analyzer, and the relative mRNA expression was calculated and counted by 2 ^-△△Ct^.

### Detection of the Protein Expression by Western Blot Analysis

Cells were collected, and the protein was extracted using a one-step animal cell active protein extraction kit (Sango Biotechnology, Shanghai, China, product No. c500022), and the protein concentration was detected using a Bradford protein detection kit (Sango Biotechnology, Shanghai, China, product No. c503031). Western blot analysis was carried out on the fully based automated system (San Jose, CA, United States). Two main antibodies were purchased: anti-ERK1 (Abcam, ab32537, 1:50 dilution) and goat anti-rabbit anti-β-tubulin (ProteinSimple, cell signaling, 2148, 1:400 dilution). We used β-tubulin as the internal parameter and prepared 100 mm dithiothreitol, 5× master mix, ladder, luminol-s, and peroxide premixed solution, respectively, according to the instructions. Then, we diluted the previously extracted protein with the 5× master mix to a solution with a final concentration of 0.2 mg/ml and denatured the solution at 95°C for 5 min. The sample and antibody were added to the board according to the instructions and tested on the computer.

### Cell Culture and Grouping

HUVECs were cultured in Dulbecco’s modified Eagle medium (DMEM) containing 10% fetal bovine serum, 100 U/mL penicillin, and 100 mg/L streptomycin. The medium was cultured in an incubator with 5% CO_2_ at 37°C and changed daily. The third to eighth generation cells were used in all experiments. The HUVECs grew to about 80% fusion. After centrifugation, 1.5 ml DMEM (4°C precooling) containing 10% dimethyl sulfoxide was added to the cell precipitation. After blowing and mixing, it was transferred to a 2-ml cell cryopreservation tube and stored in a 80°C refrigerator.

**FIGURE 2:**
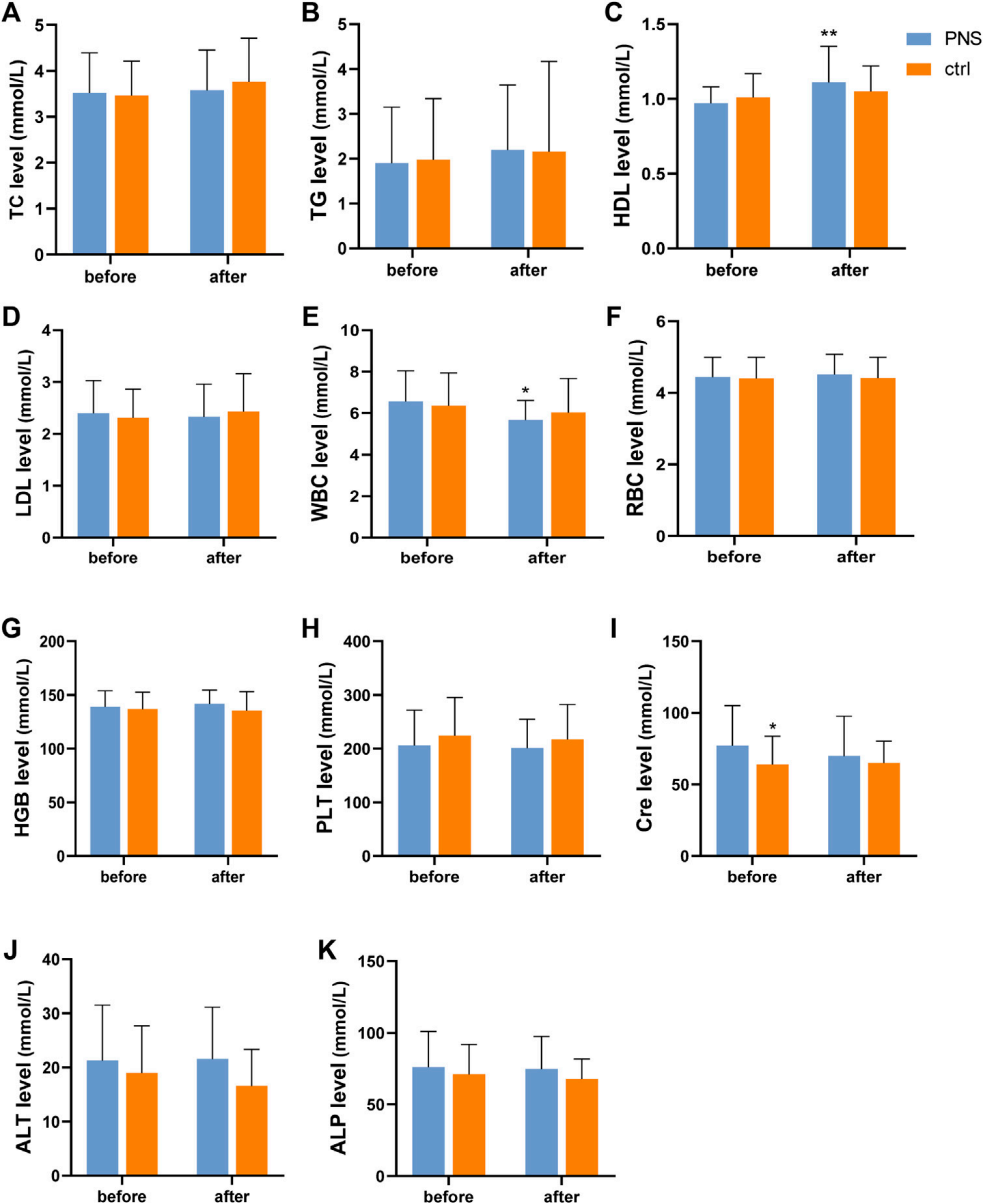
Cell experimental protocol as described in detail in the Materials and Methods section.

HUVECs were divided into three groups: a control group, an H_2_O_2_ model group, and an H_2_O_2_ model + PNS group. The H_2_O_2_ model group was treated with 100 μmol/L H_2_O_2_ for 3 h, and the H_2_O_2_ model + PNS group was treated with 100 μmol/L H_2_O_2_ for 3 h and then 30 mg/L PNS for 24 h. Cell samples were collected 24 h after treatment with H_2_O_2_ for 3 h (Figure 2). The standard of PNS was provided by the National Institutes for Food and Drug Control and included 26.3% Rg1, 27.0% Rb1, 7.4% R1, 3.7% Re, and 7.6% Rd. The concentration of PNS was 30 μg/ml.

### Annexin V/Propidium Iodide (PI) Apoptosis Assay

The HUVECs were divided into groups, as shown in Figure 2. Then, 48 h after infection with Lenti-Vector Control and Lenti-Bmi1-shRNA and 24 h after treatment with cisplatin, we centrifuged the cells at 1000 r/min for 5 min. We counted 1 × 10^6^ cells and added 100 μL of binding buffer into a 1.5 ml centrifuge tube. Then, 5 μL of annexin V was incubated, and 3 μL of PI was added. Apoptosis was analyzed by flow cytometry with 400 μL binding buffer.

### Statistical Analysis

SPSS software version 19.0 was used for statistical analysis. Data are expressed as the mean ± standard deviation. For comparisons of two groups, an independent sample *t*-test was used to analyze normal distributions, and a rank-sum test was used to analyze non-normal distributions. For multiple comparisons, the quantitative data on normal distributions were analyzed using variance analysis and a Q test, while those of non-normal distributions were analyzed using a nonparametric test.

## Results

### PNS Had an Effect Against CAD

**FIGURE 3:**
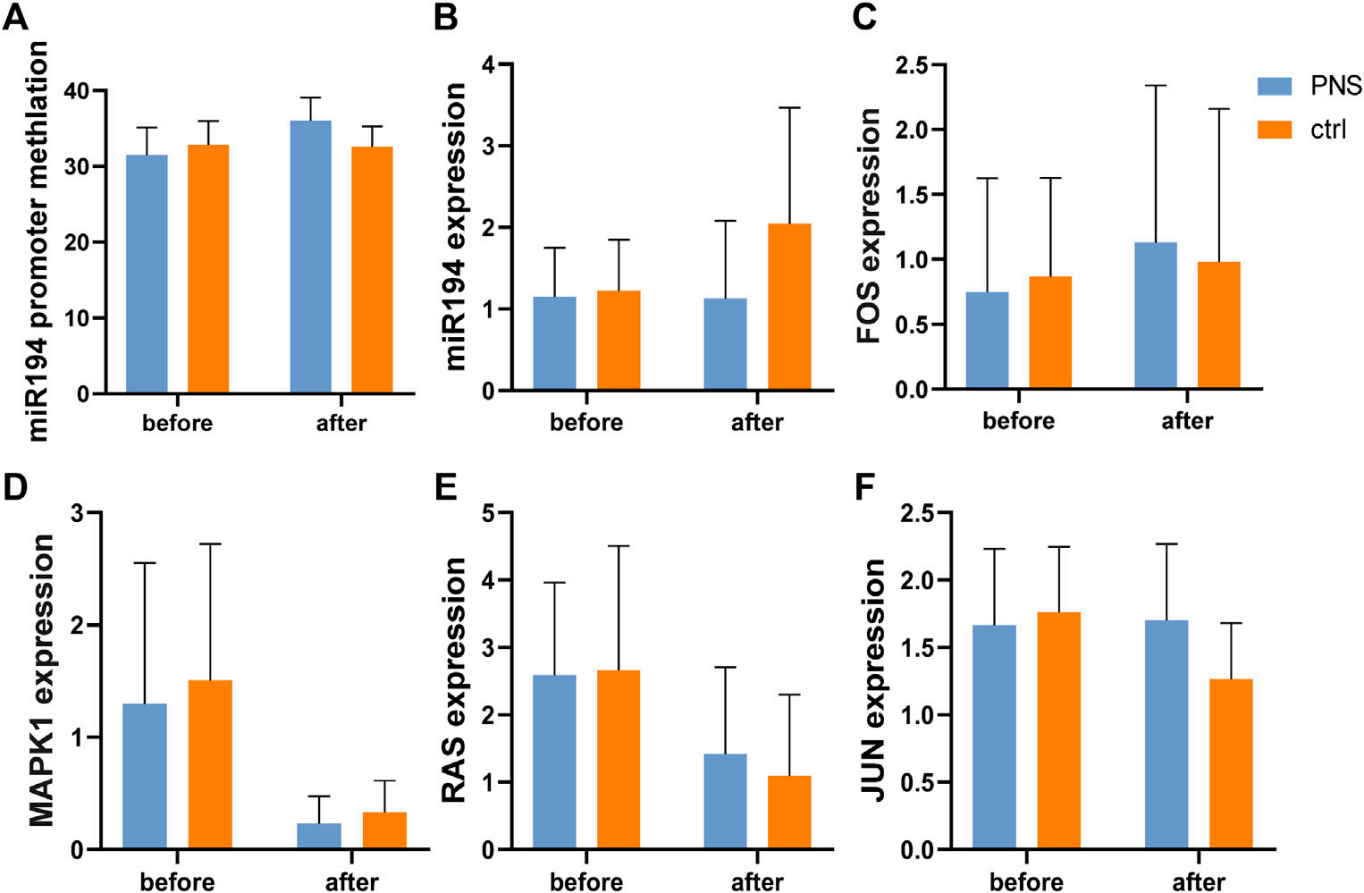
Study participant flow diagram.

This study included 95 patients with CAD. During the study period, 11 patients were shed, and 84 patients completed the study (Figure 3. Statistical analysis was conducted on the personal and clinical data on the 84 patients, including sex, age, smoking history, diabetes status, hypertension status, blood glucose, total cholesterol, and triglycerides. No significant difference was observed between the PNS and placebo groups before treatment regarding demographics, diabetes status, hypertension, blood glucose, total cholesterol, or low-density lipoprotein cholesterol (*p* > 0.05) (Table 1).

**TABLE 1:** Comparison of characteristics between PNS and the placebo group.

	PNS (n = 43)	Placebo (n = 41)
Age (years)	65.39 ± 8.68	63.63 ± 7.05
Sex (male, %)	28 (65%)	32 (78%)
Smoking history [n (%)]	5 (12%)	5 (12%)
Hypertension [n (%)]	22 (51%)	26 (63%)
Diabetes [n (%)]	17 (40%)	16 (39%)
TC (mmol/L)	3.63 ± 0.90	3.65 ± 0.68
TG (mmol/L)	1.95 ± 1.26	2.25 ± 1.48
LDL-C (mmol/L)	2.65 ± 0.87	2.52 ± 0.75
HDL-C (mmol/L)	0.99 ± 0.16	1.04 ± 0.51
Aspirin administration [n (%)]	31 (72%)	28 (68%)
Statin administration [n (%)]	28 (65%)	29 (70%)

TC: total cholesterol; TG: triglycerides; LDL-C: low-density lipoprotein cholesterol; HDL-C: high-density lipoprotein cholesterol.

We also observed the incidence of cardiovascular adverse events within 60 days after treatment. No difference was observed between the two groups (Table 2).

**TABLE 2:** Adverse cardiovascular events 60 days after treatment between PNS and the placebo group.

	PNS (n = 43)	Placebo (n = 41)
Death [n (%)]	0 (0%)	0 (0%)
Myocardial infarction [n (%)]	0 (0%)	0 (0%)
Stroke [n (%)]	0 (0%)	0 (0%)
Readmission due to angina [n (%)]	2 (5%)	2 (5%)

Questionnaires were filled out on the day of admission and 4 weeks after treatment to calculate the occurrence of angina attacks in the two groups. The length of angina attacks was significantly reduced in both groups. However, treatment with PNS led to a significantly greater reduction than the placebo. Additionally, both groups had a significant decrease in the frequency of angina attacks after treatment, although no significant difference was observed in the groups (Table 3A, Table 3B).

**TABLE 3:** (Panel A) Length of angina attacks; (Panel B) Frequency of angina attacks.

Panel A	N	Before (min/time)	After (min/time)
PNS	41	8.95 ± 1.13	2.50 ± 1.26^△△^
Placebo	43	8.53 ± 1.36	4.33 ± 1.05**^##^

Panel B	n	Before (times/wk)	After (times/wk)
PNS	41	7.15 ± 4.23	1.24 ± 1.08^△^
Placebo	43	8.24 ± 5.15	2.14 ± 1.26*

^△^ *p* < 0.05 and ^△△^ *p* < 0.01, significantly different from the PNS group before. *p < 0.05 and **p < 0.01, significantly different from the placebo group before. ^#^ *p* < 0.05 and ^##^ *p* < 0.01, significantly different from the PNS group after.

**FIGURE 4:**
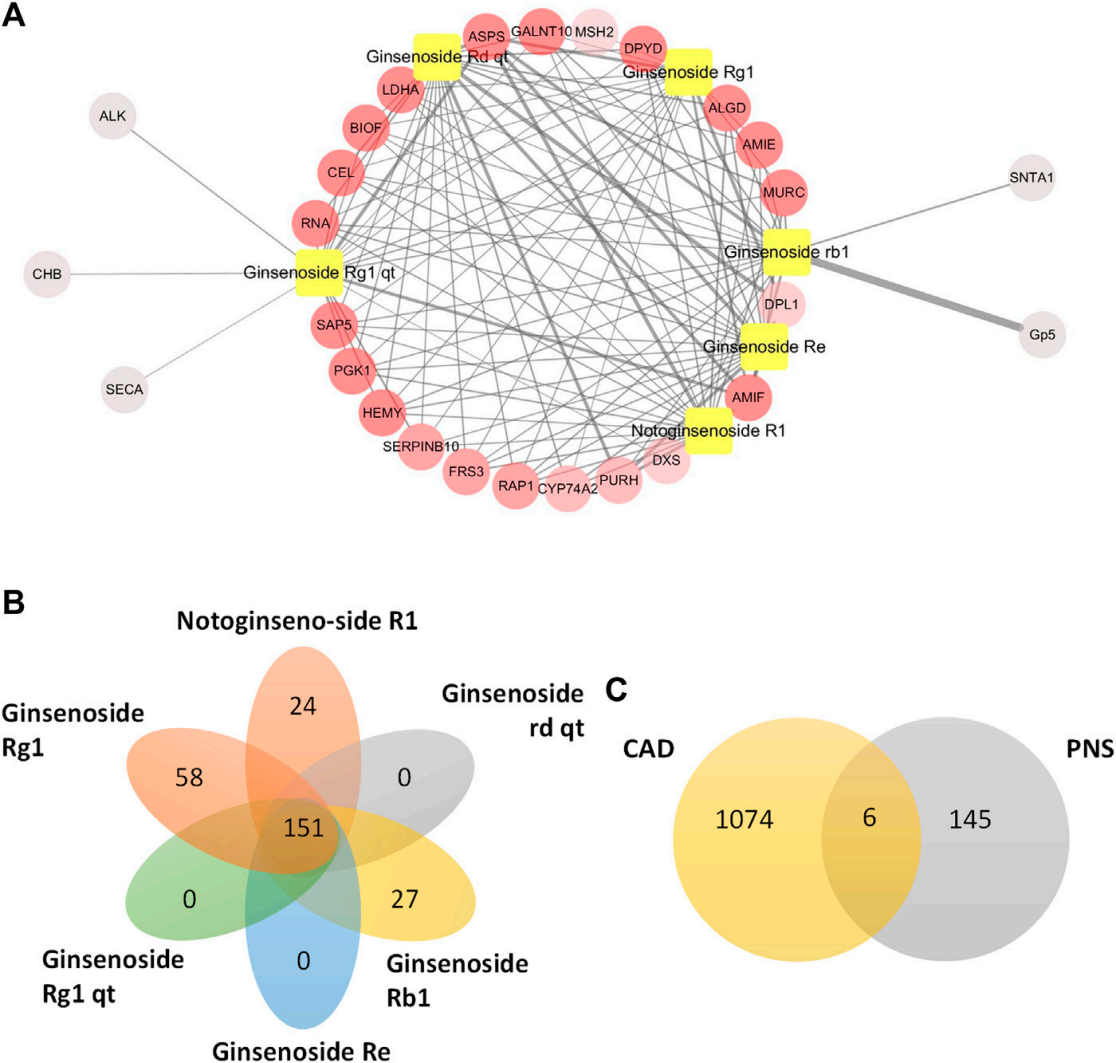
Blood tests before and after treatment in the PNS and placebo groups. *p* < 0.05 and **p < 0.01, significantly different from the PNS group before.

Routine blood and biochemical examination were measured on days 1 and 30 after enrollment. HDL significantly increased (*p* < 0.01) after treatment in the PNS group (Figure 4C). In other aspects of blood lipids, no significant difference was observed between the two groups before and after treatment (Figure 4A,B,D). Additionally, the white blood cell (WBC) count decreased significantly after PNS treatment, which confirmed the anti-inflammatory effect of PNS (Figure 4E). Meanwhile, hemoglobin (HGB) levels increased in the PNS group and decreased in the placebo group (Figure 4G). Kidney and liver functions were similar before and after treatment in the two groups (Figure 4I–K). However, the Cre level decreased in the PNS group (Figure 4I).

### Prediction of PNS Targets

**FIGURE 5:**
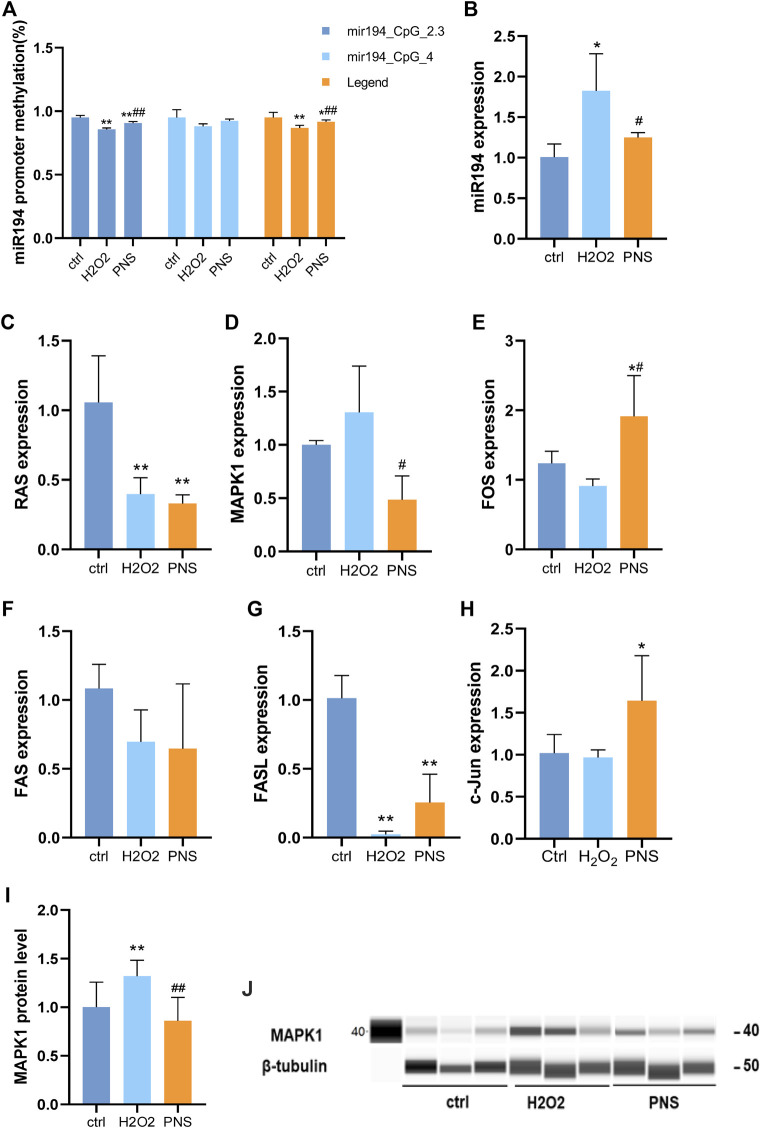
Prediction of PNS targets by pharmacological analysis. **(A)** Top 20 potential targets of PNS. The relationship of the top 20 best matching genes of PNS was exhibited by Cytoscape. Yellow squares indicate the main ingredients of PNS. The circles indicate the potential targets. The nodes with a higher degree appear redder in color. The nodes with a higher fit value have thicker edges. **(B)** Common targets of the main ingredients of PNS. **(C)** Common targets of PNS potential targets and CAD-related genes.

Five main active ingredients in PNS were identified in the Chinese Pharmacopoeia (2015 edition), namely, notoginsenoside R1, ginsenoside Rg1, ginsenoside Re, ginsenoside Rd, and ginsenoside Rb1. Then, the Traditional Chinese Medicine Systems Pharmacology Database and Analysis Platform were used to evaluate the pharmacokinetic-related properties of PNS. Through the PharmMapper database, potential targets of the five active ingredients were predicted. Then, 4 ml venous blood from patients with CAD and controls was collected and placed in EDTA tubes. DNA was extracted from centrifuged PBMCs. We sequenced the captured bisulfite transformed DNA samples using Illumina HiSeq2500 and Roche SeqCap Epi methylation enrichment kits. Then, we found genes related to CAD and identified the top 300 potential targets of every active component. We obtained 429 targets of the six molecules and found that these six main ingredients of PNS shared some common targets. The top 20 potential targets were extracted according to their fit value. Notably, the potential targets of each molecule were closely interrelated (Figure 5A). A total of 151 common targets of the main ingredients of PNS were selected for further investigation (Figure 5B). Six genes were common targets of PNS in CAD sequencing, namely, *NOTCH1*, *ECE1*, *SOCS3*, *MAPK1*, *RAS*, and Fas cell surface death receptor ligand (FASL) (Figure 5C).

### PNS Changes Hypomethylation of miR-194

**FIGURE 6:**
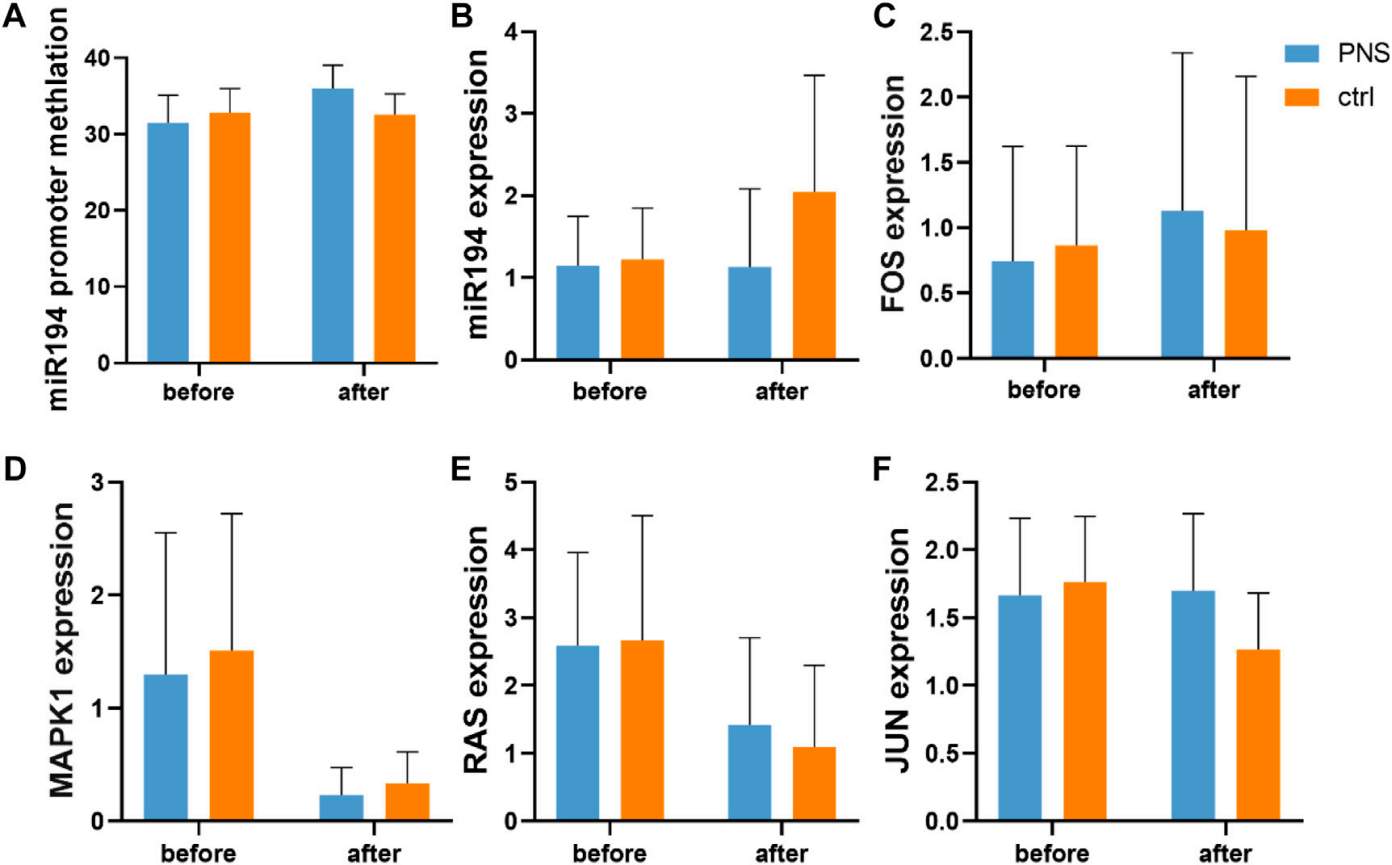
Expression of key genes in the miR-194 promoter-miR914-MAPK pathway according to pyrosequencing and PCR.

According to the gene test, the level of methylation of miR-194 was similar before treatment in the two groups, and after treatment, no significant difference was observed (Figure 6A). However, the level of DNA methylation in the miR-194 promoter region increased in the PNS group after treatment but not in the placebo group.

The expression levels of the key nodes in the PNS and placebo groups were obtained using qRT-PCR and compared. No significant difference was observed between the two groups before and after treatment. However, while no significant difference in the expression of miR-194 was observed in the PNS group after treatment, that of the placebo group increased (Figure 6B–E). In addition, there was no significant difference between mRNA RAS, Fos proto-oncogene (FOS), MAPK1, and Jun proto-oncogene (c-Jun) after treatment.

### Effects of PNS on DNA Methylation, miRNA, and Target Genes Related to Oxidative Damage in HUVECs

**FIGURE 7:**
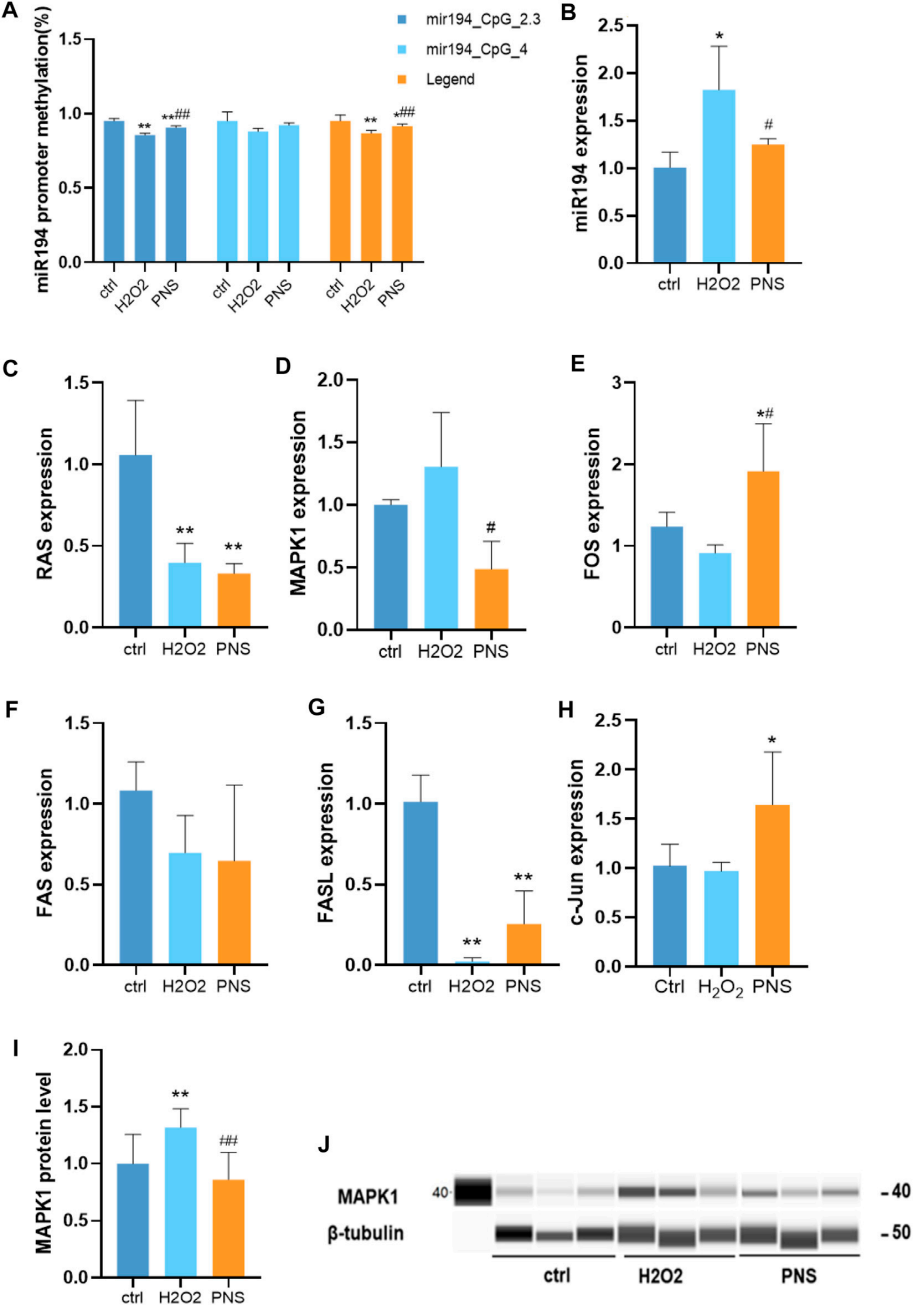
Expression of key genes in the miR-194 promoter-miR-194-MAPK pathway by MSPCR, qRT-PCR, and Western blotting *in vitro* **(A)** DNA methylation level of the miR-194 promoter by MSPCR; **(B)** miR-194 expression; **(C)**RAS expression; **(D)** MAPK1 expression; **(E)** FOS expression; **(F)** FAS expression; **(G)** FASL expression; **(H)** c-Jun expression; **(I)** MAPK1 protein level; and **(J)** MAPK1 protein.

The methylation level always was changed in the early stage of life or by the influence of a long time in general. However, PNS could change the trend of methylation. We observed that miR-194 in HUVECs was hypermethylated by H_2_O_2_, and miR-194 was hypomethylated by PNS treatment (Figures 7A, 4B). Then, by qRT-PCR, we detected the expression level of miR-194 and found that it was upregulated in H_2_O_2_ HUVECs and downregulated in PNS-treated cells, which was the opposite trend of miR-194 methylation (Figure 7B). Thus, hypomethylation of the gene would lead to overexpression, and hypermethylation would inhibit expression, which was consistent with normal results of methylation.

Compared with the H_2_O_2_ group, MAPK1 was upregulated, and FAS, c-Jun, RAS, FOS, and FASL were downregulated (Figure 7C–G). However, after PNS intervention, MAPK1 expression was significantly downregulated, while FOS and FASL expressions were upregulated compared to H_2_O_2_-induced HUVECs. The results of PCR conformed to the targets of the pharmacological analysis, MAPK1, FASL, and RAS.

In addition, we performed Western blot analysis to detect the MAPK1 expression. The results generally resembled those of the mRNA expression of the key genes. In the H_2_O_2_ group, MAPK1 was upregulated; however, after PNS intervention, MAPK1 was downregulated compared to the levels in H_2_O_2_-induced HUVECs.

### PNS Protects HUVECs From Apoptosis by H_2_O_2_ Induction

**FIGURE 8:**
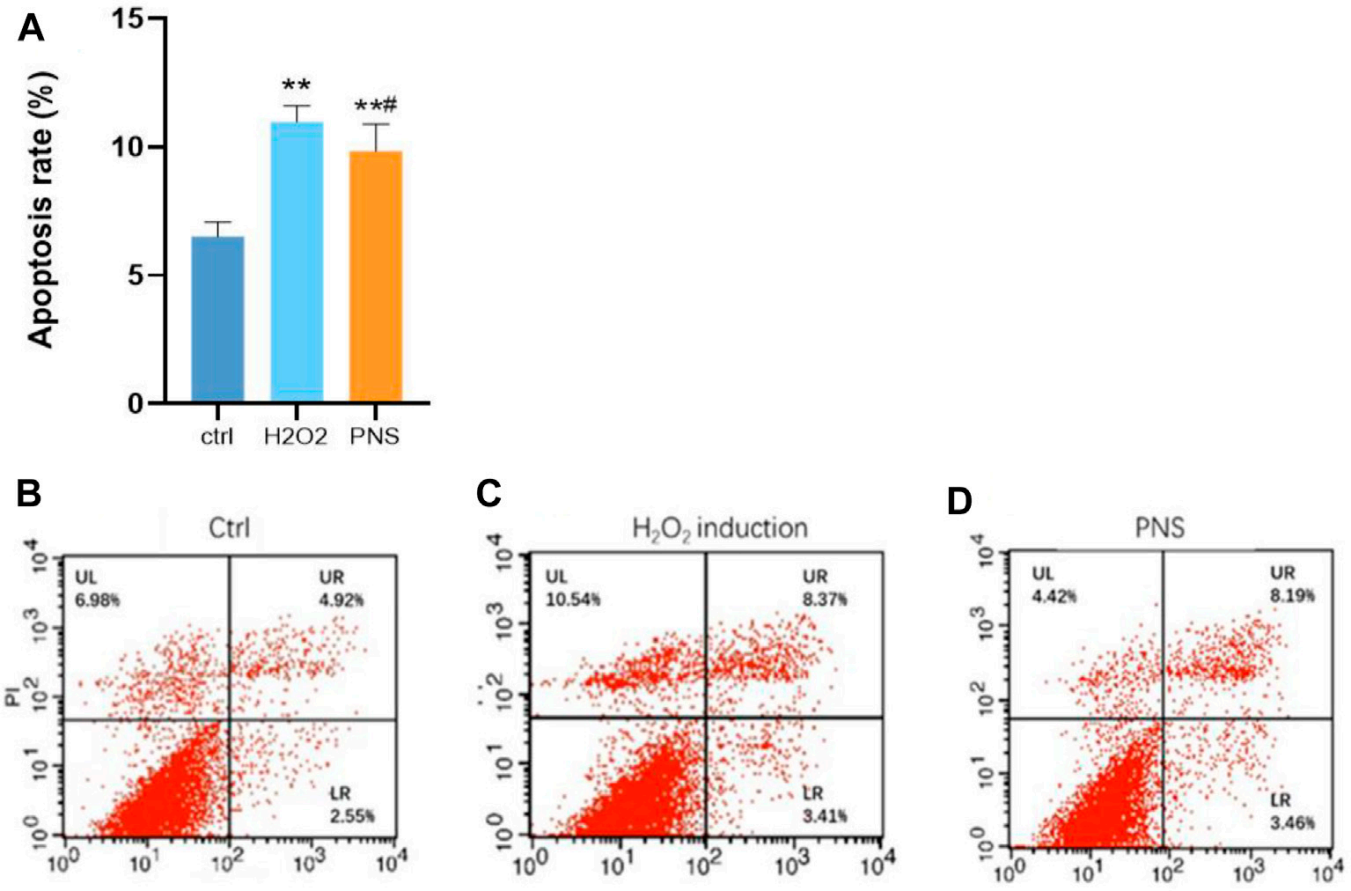
Annexin V/PI apoptosis assay results. **(A)** Level of apoptosis in HUVECs; **(B)** control group; **(C)** H_2_O_2_ group; and **(D)** PNS group. *p < 0.05 and **p < 0.01, significantly different from the control group. ^#^ *p* < 0.05, significantly different from the H_2_O_2_ group.

Using annexin V/PI apoptosis assay, we observed that apoptosis was differentially upregulated in oxidative HUVECs (Figure 8A). With the intervention of PNS, the apoptosis rate significantly decreased. Presumably, PNS plays an important role in damaged cells during the early stages of injury (Figure 8A–D).

## Discussion

According to previous research, a randomized controlled trial was conducted to observe the clinical effects and changes in key nodes of the miR-194 promoter-miR-194-MAPK signaling pathway. A total of 84 patients with CAD were collected and randomly divided into two groups (the PNS group and placebo group). In terms of the length of angina pectoris attacks, both groups demonstrated a significant decrease after treatment, and the PNS group had a larger reduction. In the comparison of blood lipids, HDL changed significantly after treatment in the PNS group. We previously conducted a meta-analysis including 17 studies with a total of 2,315 patients with UA. We found that PNS had a promising therapeutic effect on the reduction of the primary end point, frequency, and duration of angina attacks in patients with UA (Yang et al., 2014; Duan et al., 2018).

In our previous research, we identified a unique DNA methylation-miRNA-mRNA regulatory network for CAD. We also identified key signaling pathways in this network, including the miR-194 promoter-miR-194-MAPK signaling pathway in CAD by pyrosequencing, MSPCR, qRT-PCR, and Western blotting analysis. Moreover, miR-194 is one of the hepatic-enriched miRNAs. In addition, it is upregulated in the serum of patients with myocardial infarction and is closely correlated with impaired cardiac function (Matsumoto et al., 2013). In addition, miR194 was increased in a lipopolysaccharide-induced H9c2 cardiomyocyte injury model. It also promoted cardiomyocyte apoptosis and participated in myocardial injury induced by endotoxemia (Zhai and Ding, 2018). The upregulation of circulating miR194 levels was found to be closely correlated with impaired human cardiac function, including ejection fraction and NT-proBNP levels. In addition, miR194 sponges could improve obesity-mediated cardiac dysfunction *in vivo* (Nie et al., 2018). The expression of miR194 degraded coronary vascular tissues and endothelial cells of rats with atherosclerosis (Li et al., 2021). Meanwhile, silencing miR-194-5p could alleviate doxorubicin-induced cardiotoxicity *via* PAK2 *in vitro* and *in vivo*. Moreover, inhibition of miR-194-5p or the overexpression of PAK2 reduced DOX-induced apoptosis of cardiomyocytes. Silencing of miR-194-5p also improved DOX-induced cardiac dysfunction (Fa et al., 2022). Moreover, activation of 5′AMP-activated protein kinase (AMPK)-p21-activated kinase 2 (PAK2) signaling attenuated ER stress and myocardial apoptosis induced by ischemia/reperfusion injury (Xu et al., 2020). Finally, the phosphorylation level of MAPK was elevated by miR-194 (Latouche et al., 2016; Bai et al., 2017; Niu et al., 2018; Liu et al., 2021). Similarly, in our cell experiment, miR-194 was upregulated in H_2_O_2_-induced HUVECs and decreased by PNS treatment.

No significant difference was observed in miR-194 levels in the PNS group after treatment; however, in the placebo group, these levels increased. In addition, no significant difference was observed in mRNA RAS, FOS, MAPK1, or c-Jun after treatment. However, RAS expression decreased after treatment, and the decrease was more obvious in the placebo group. Moreover, FOS in the PNS group had an increasing trend, but the placebo group did not. MAPK1 decreased after treatment, but there were no differences between the two groups. In addition, c-Jun did not change significantly in the PNS group, while in the placebo group, there was a significant downward trend.

We found that PNS can improve the symptoms of patients with CAD. In addition, we observed that PNS in traditional Chinese medicine affects DNA methylation-miRNA-mRNA. Using pharmacological analysis, some of the PNS targets were found to be differentially expressed genes in CAD sequencing. Six genes were common targets of PNS in CAD sequencing, including NOTCH1, ECE1, SOCS3, MAPK1, RAS, and FASL. Additionally, genes in the interaction network and clinical parameters were correlated. Meanwhile, *in vitro* experiments demonstrated that the key nodes in the miR-194 promoter-miR-194-MAPK signaling pathway in CAD are changed by PNS. A previous study reported that PNS conferred profound protection through the downregulation of MAPK (Luo et al., 2021; Yang et al., 2022). Finally, intervention with PNS is likely to improve apoptosis.

The present study has several limitations. First, the sample size of the randomized controlled trial was relatively small. Second, the samples came from the same hospital, and using samples from different regions may be beneficial. Third, while miR-194 and its target gene demonstrated a trend from PNS treatment, the change was not significant, likely because the intervention time with PNS was not long enough. In the future, a larger, multicenter study with a longer intervention period is warranted to confirm the clinical efficacy of PNS.

In conclusion, in a randomized controlled clinical trial, PNS alleviated CAD and changed the key nodes of the miR-194 promoter-miR-194-MAPK signaling pathway. PNS also acted on this pathway *in vitro*. PNS are important for the intervention of CAD, and the miR-194 promoter-miR-194-MAPK signaling pathway is likely to be a therapeutic target of PNS for CAD.

## Data Availability

The original contributions presented in the study are included in the article/supplementary material; further inquiries can be directed to the corresponding author.

## Abbreviations

CAD, coronary artery disease; c-Jun, Jun proto-oncogene; DMEM, Dulbecco’s modified Eagle medium; DBP, diastolic blood pressure; ECG, electrocardiogram; FASL, Fas cell surface death receptor ligand; FOS, Fos proto-oncogene; HGB, hemoglobin; HDL, high-density lipoprotein; LDL, low-density lipoprotein cholesterol; MAPK, mitogen-activated protein kinase; miRNA, microRNA; MSPCR, methylation-specific PCR; PNS, *Panax notoginseng* saponins; PI, propidium iodide; qRT-PCR, quantitative real-time PCR; RAS, rat sarcoma; SBP, systolic blood pressure; TCM, traditional Chinese medicine; TC, total cholesterol; TG, triglycerides; UA, unstable angina; WBC, white blood cell.
